# Triglyceride glucose index and Atherogenic index of plasma for predicting colorectal neoplasms in patients without cardiovascular diseases

**DOI:** 10.3389/fonc.2022.1031259

**Published:** 2022-11-14

**Authors:** Muzhou Han, Hao Wang, Shuyue Yang, Siying Zhu, Guiping Zhao, Haiyun Shi, Peng Li

**Affiliations:** ^1^ Department of Gastroenterology, Beijing Friendship Hospital, Capital Medical University, National Clinical Research Center for Digestive Diseases, Beijing Digestive Disease Center, Beijing, China; ^2^ Department of Clinical Epidemiology and Evidence-based Medicine, Beijing Friendship Hospital, Capital Medical University, Beijing, China

**Keywords:** triglyceride glucose index, Atherogenic index of plasma, colorectal neoplasms, cardiovascular disease, insulin resistance

## Abstract

**Background and aims:**

Colorectal neoplasms (CRN) include colorectal cancer (CRC) and colorectal adenoma (CRA). The relationship between CRN and triglyceride-glucose (TyG) index or between CRN and atherogenic index of plasma (AIP) is unclear. This study aims to investigate the roles of TyG index and AIP in predicting CRN in people without cardiovascular disease (CVD).

**Methods:**

2409 patients without CVD underwent colonoscopy were enrolled. Clinical information and relevant laboratory test results of these patients were collected and recorded. According to endoscopic and pathological results, all participants were divided into a neoplasms group and a non-neoplasms group. The TyG index was calculated as ln (TGs×FPG/2), while AIP was calculated as log (TGs/HDL-C). We used uni- and multivariate logistic regression and restricted cubic spline (RCS) to analyze the association between the TyG inedx, AIP and CRN, develop predictive models and construct the nomograms. Receiver operating characteristic (ROC) curves were utilized to evaluate the predictive value for CRN.

**Results:**

Participants in the neoplasms group were more likely to be older, have higher TyG index, higher AIP and higher rates of fecal occult blood test positivity, and were more likely to be male, smokers and those with the family history of CRC (*P* < 0.05). The higher TyG index was related to the higher risk of CRN [OR (95% CI): 1.23 (1.08 - 1.41), *P* = 0.003]. The higher AIP was related to the higher risk of CRN [OR (95% CI): 1.55 (1.16 - 2.06), *P* = 0.003]. These two indicators are better for predicting CRN in women than men. The combined use of the TyG index and other independent risk factors (age, sex, smoking status, family history and FOBT) to distinguish CRN was effective, with a sensitivity of 61.0%, a specificity of 65.1% and an AUC of 0.669 (95%CI, 0.639 - 0.698). Likewise, the combined use of the AIP and other independent risk factors to distinguish CRN was also effective, the model had an overall 56.3% sensitivity and 68.7% specificity with an AUC of 0.667 (95%CI, 0.638 - 0.697).

**Conclusion:**

This study showed that the TyG index and the AIP might be biomarkers that could be used to predict the risk of CRN in patients without CVD.

## Introduction

Colorectal cancer (CRC) is recognized as the world’s fourth most deadly cancer with almost 900,000 deaths annually (1). Colorectal adenoma (CRA) is usually regarded to be precursor lesion for most CRC (2, 3). Both are collectively referred to as colorectal neoplasms (CRN), which are major public health problems that result in significant economic burdens for governments. There is an urgent need for usable and reliable non-invasive markers to make an early diagnosis of patients at risk of colorectal neoplasms in the general population, and then encourage those at risk to undergo a colonoscopy to reduce CRC morbidity and mortality (4).

Recently, the triglyceride-glucose (TyG) index and the atherosclerotic index of plasma (AIP) have attracted extensive attention and medical research. The TyG index, a parameter derived from the fasting blood glucose and triglyceride levels, has been evaluated as a reliable surrogate for insulin resistance (IR) (5–7). Numerous studies have shown that TyG index is an autonomous risk factor for the incidence of cardiovascular disease (CVD) (8–11). It is also associated with an increased risk of diabetes (12), hypertension (13) and nonalcoholic fatty liver disease (14). The atherogenic index of plasma (AIP) is the logarithmically transformed ratio of triglyceride (TG) to high-density lipoprotein-cholesterol (HDL-C) in molar concentration (mmol/L) (15). It has been proven to be a reliable biomarker for the prediction of various vascular diseases (16). AIP is also closely related to insulin resistance, diabetes and metabolic syndrome (17–19). Although these two indicators have demonstrated an outstanding performance in diagnosing CVD and metabolic disease, the association between either TyG index or AIP and CRN still needs further exploration. To date few relevant studies have been scheduled to investigate this correlation.

Considering that the TyG index and AIP are closely related to CVD, we chose to explore the role of these two indicators in predicting CRN in those people without CVD. We chose this approach in order to exclude the effect of CVD on these two indicators.

## Methods

### Participants

**Figure 1:**
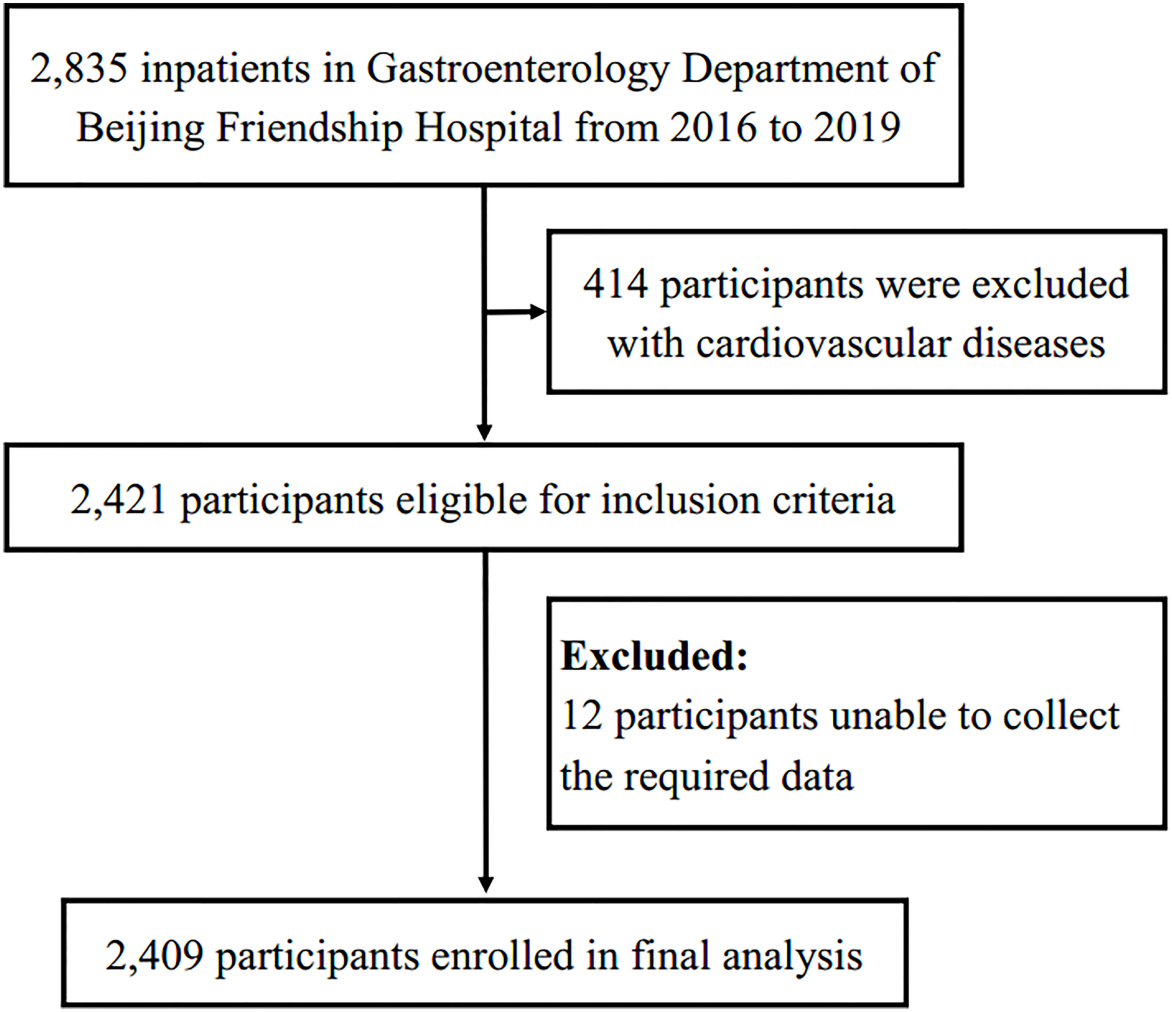
Flow chart of the study population.

This is a single-center retrospective analysis of 2835 patients (aged ≥ 18 years) that were admitted to Beijing Friendship Hospital. They underwent a colonoscopy during the period January 1, 2016 to December 31, 2019. Participants with a reported previous history of cardiovascular diseases (n=414) and participants with missing baseline data (n=12) were excluded. In total, 2409 participants were finally included in the current study (**Figure 1**). This study was in accordance with the principles of the Declaration of Helsinki and approved by the ethics committee of Beijing Friendship Hospital, Capital Medical University (No.2021-P3-138-01). Given the retrospective nature of the present research, no informed consent was required.

### Data collection and definitions

Data of demographic and clinical characteristics, including sex, age, body mass index (BMI), history of smoking and drinking, and family history of CRC were extracted from the electronic medical recording system of the Beijing Friendship Hospital. On admission, blood samples from the median cubital vein were collected early in the morning after an overnight fast (> 8 h). We analyzed fasting triglyceride (TGs), fasting plasma glucose (FPG) and high-density lipoprotein-cholesterol (HDL-C) levels immediately after sampling. Fecal samples were taken for fecal occult blood testing (FOBT) within 24 hours before the scheduled colonoscopy. The TyG index was calculated as ln [fasting TGs (mg/dL)×FPG (mg/dL)/2] (20). AIP was analytically calculated from log (TGs/HDL-C), where TGs and HDL-C were expressed in millimole per liter (15). CVD are defined as pathological conditions involving the cardiovascular system including the heart, the blood vessels, or the pericardium.

A colonoscopy was performed by endoscopists that had performed a minimum of 500 colonoscopies. All detected polyps were biopsied or removed. The histopathology was determined by experienced gastrointestinal pathologists. According to endoscopic and pathological results, all participants were divided into a neoplasms group and a non-neoplasms group. The neoplasms group consisted of patients with CRC or CRA. The non-neoplasms group consisted of patients without any evidence of elevated lesions under endoscopy, as well as patients with non-adenomatous colorectal polyps.

### Statistical analysis

Normally distributed continuous variables were reported as mean ± standard deviation (SD), and categorical variables were represented as frequencies and percentages. The Student *t-test* was applied for the continuous variables and the *Chi-square* test was applied for categorical variables to compare the differences between the two groups. Univariate and multivariate logistic regression models were used to evaluate the associations between different indices and neoplasms in non-CVD participants. Model 1: adjusted for age; Model 2: adjusted for age and sex; Model 3: adjusted for age, sex, family history and FOBT. The restricted cubic splines with 4 knots were applied to determine the nonlinear relationship between different indices and the risk of neoplasms in non-CVD participants. A multivariate binary logistic regression was used to construct diagnostic models for neoplasms. Receiver operating characteristic (ROC) curves and the area under the curve (AUC) were used to assess the diagnostic performance of the models. To facilitate the clinical application of diagnostic model 3, a nomogram was developed based on AIP, TyG and other potential risk factors of neoplasms. A data analysis was performed using SPSS 25.0 (IBM Corporation, New York, USA) and R version 4.1.2 (R core Team 2017). All reported *P* values were two-tailed, and *P* < 0.05 was considered to be statistically significant.

## Results

### Baseline characteristics

The 2409 participants (56.25% men, mean age 57.18 ± 11.26 years) were divided into a non-neoplasms group (n=947) and a neoplasms group (n=1462). Baseline characteristics of the total population and groups are presented in **Table 1**. Participants in the neoplasms group were more likely to be older, have higher TyG index and AIP, and have higher rates of fecal occult blood test (FOBT) positivity. They were also more likely to be male, smokers and have a family history of colorectal cancer (*P* < 0.05).

**Table 1:** Characteristics of the study participants.

Characteristics	Total (n = 2409)	Non-neoplasm (n = 947)	Neoplasm (n = 1462)	*P* value
Sex, n (%)				<0.001
Male	1355 (56.25)	457 (48.26)	898 (61.42)	
Female	1054 (43.75)	490 (51.74)	564 (38.58)	
Age, years	57.18 ± 11.26	54.04 ± 11.87	59.22 ± 10.36	<0.001
BMI, n (%)				0.938
<30 kg/m^2^	2245 (93.19)	883 (93.24)	1362 (93.16)	
≥30 kg/m^2^	164 (6.81)	64 (6.76)	100 (6.84)	
Smoking status, n (%)				<0.001
Smokers	609 (25.28)	183 (19.32)	426 (29.14)	
Non-smoker	1800 (74.72)	764 (80.68)	1036 (70.86)	
Drinking status, n (%)				0.115
Drinkers	523 (21.71)	190 (20.06)	333 (22.78)	
Non-drinkers	1886 (78.29)	757 (79.94)	1129 (77.22)	
FPG, mmol/L	5.25 ± 1.19	5.24 ± 1.13	5.26 ± 1.23	0.804
TC, mmol/L	4.76 ± 1.04	4.71 ± 1.13	4.79 ± 0.97	0.139
TG, mmol/L	1.70 ± 1.45	1.65 ± 1.61	1.73 ± 1.34	0.211
HDL-C, mmol/L	1.13 ± 0.29	1.14 ± 0.31	1.13 ± 0.28	0.292
LDL-C, mmol/L	2.79 ± 1.00	2.76 ± 1.36	2.82 ± 0.68	0.201
TyG index	8.68 ± 0.61	8.63 ± 0.63	8.71 ± 0.60	0.003
AIP index	0.12 ± 0.29	0.09 ± 0.30	0.13 ± 0.28	0.003
Family History, n (%)	145 (6.02)	34 (3.59)	111 (7.59)	<0.001
FOBT, n (%)	126 (5.23)	29 (3.06)	97 (6.63)	<0.001

Data are presented as medians ± standard deviation or frequencies (percentages). P < 0.05 is considered statistically significant as for the differences between the particular medians of hypertension group and polyps group. n, number of participants; BMI, body mass index; FPG, fasting plasma glucose; TC, total cholesterol; TG, total triglycerides; HDL-C, high-density lipoprotein cholesterol; LDL-C, low-density lipoprotein cholesterol; TyG index, triglyceride–glucose index; AIP, atherogenic index of plasma; FOBT, fecal occult blood test; CVD, cardiovascular disease.

### Associations between TyG index, AIP and neoplasm

As shown in **Table 2**, as a ranking variable, the TyG index in the highest quartile (the lowest quartile as the reference) was associated with a higher risk of CRN [OR (95% CI): 1.38 (1.09 - 1.73), *P* = 0.007]. As a continuous variable, the higher TyG index was independently related to the higher risk of CRN [OR (95% CI): 1.23 (1.08 - 1.41), *P* = 0.003]. In multi-variable adjusted models, the associations between TyG index and any CRN did not attenuate. Similarly, the higher AIP was related to the higher risk of CRN [OR (95% CI): 1.55 (1.16 - 2.06), *P* = 0.003], and the association between AIP and CRN was still strong (*P* < 0.05) with an increase in other risk factors (age, sex, family history and FOBT). In the age-adjusted model, individuals in the highest quartile of AIP had a higher risk of developing CRN [OR (95% CI): 1.36 (1.08 - 1.73), *P* = 0.010] when the lowest quartile of AIP was used as a reference.

**Table 2:** Logistic regression analyses of associations between TyG, AIP index and neoplasm.

	Univariate analysis		Model 1		Model 2		Model 3	
	OR (95% CI)	*P* value	OR (95% CI)	*P* value	OR (95% CI)	*P* value	OR (95% CI)	*P* value
TyG Q1	Ref	–	Ref	–	Ref	–	Ref	–
TyG Q2	1.21 (0.96 - 1.52)	0.102	1.22 (0.96 – 1.55)	0.098	1.17 (0.92 – 1.49)	0.200	1.24 (0.94 – 1.63)	0.129
TyG Q3	1.31 (1.04 - 1.65)	0.021	1.24 (0.98 – 1.57)	0.076	1.18 (0.93 – 1.50)	0.186	1.22 (0.93 – 1.60)	0.151
TyG Q4	1.38 (1.09 - 1.73)	0.007	1.43 (1.13 – 1.82)	0.003	1.28 (1.01 – 1.63)	0.044	1.35 (1.02 – 1.77)	0.035
TyG*	1.23 (1.08 - 1.41)	0.003	1.26 (1.09 – 1.45)	0.001	1.18 (1.03 – 1.36)	0.022	1.19 (1.01 – 1.40)	0.038
AIP Q1	Ref	–	Ref	–	Ref	–	Ref	–
AIP Q2	1.28 (1.02 – 1.61)	0.033	1.22 (0.97 – 1.55)	0.095	1.14 (0.90 – 1.45)	0.278	1.07 (0.81 – 1.41)	0.637
AIP Q3	1.49 (1.18 – 1.88)	0.001	1.45 (1.14 – 1.84)	0.002	1.28 (1.01 – 1.64)	0.046	1.22 (0.92 – 1.61)	0.163
AIP Q4	1.24 (0.99 – 1.57)	0.062	1.36 (1.08 – 1.73)	0.010	1.17 (0.92 – 1.49)	0.203	1.14 (0.87 – 1.50)	0.352
AIP*	1.55 (1.16 - 2.06)	0.003	1.83 (1.36 – 2.46)	<0.001	1.51 (1.11 – 2.04)	0.008	1.43 (1.01 – 2.02)	0.045

TyG, triglyceride–glucose index; AIP, atherogenic index of plasma; TyG*, the OR was estimated by per unit increase of TyG; AIP*, the OR was estimated by per unit increase of AIP;

Model 1: adjusted for age; Model 2, adjusted for age and sex; Model 3, adjusted for age, sex, family history and FOBT.

P < 0.05 is considered statistically significant.

### Restricted cubic spline

**Figure 2:**
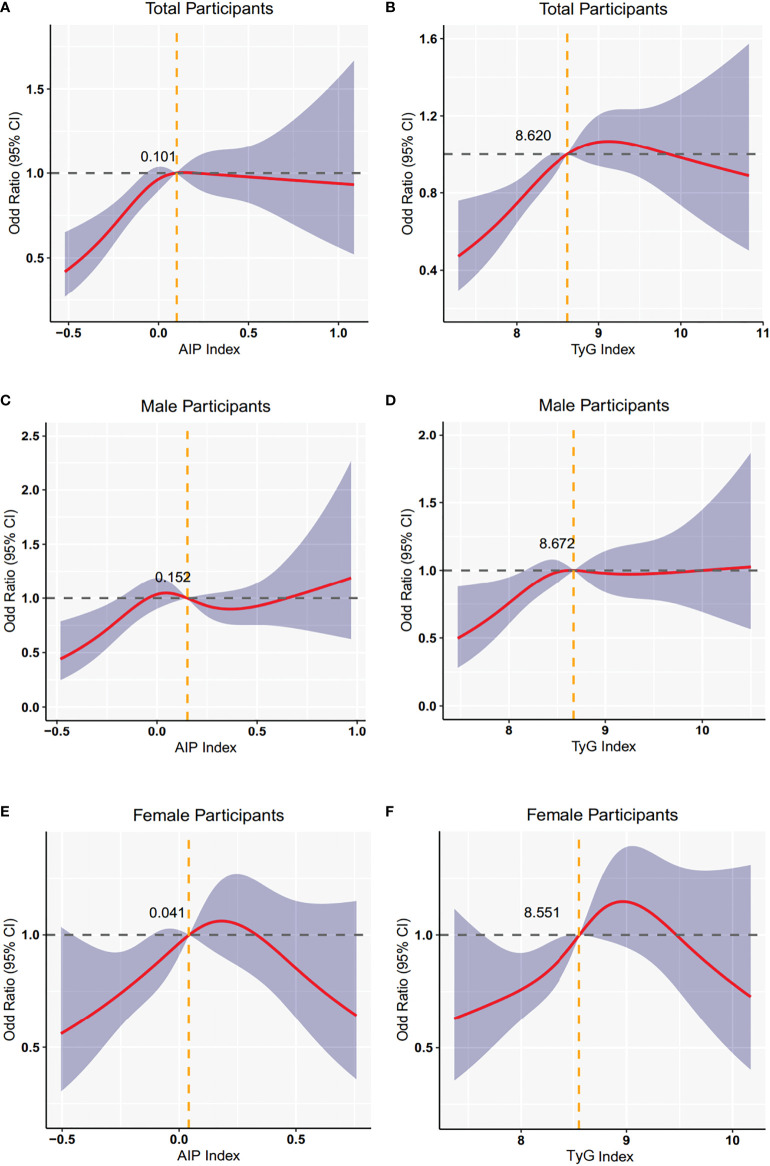
Restricted cubic spline plot of the association between TyG, AIP index and neoplasm in non-CVD participants. **(A)** the association between AIP and neoplasm in total participants. **(B)** the association between TyG index and neoplasm in total participants. **(C)** the association between AIP and neoplasm in male participants. **(D)** the association between TyG index and neoplasm in male participants. **(E)** the association between AIP and neoplasm in female participants. **(F)** the association between TyG index and neoplasm in female participants.

In **Figure 2**, we used the restricted cubic splines to visualize the relationship between TyG index, AIP and CRN across the different sexes. Within the normal AIP range, female individuals with a higher AIP had an increased prevalence of CRN when the AIP value was set to 0.041, as a reference. Analogously, in the normal TyG range, when a TyG value of 8.551 was used as a reference, female individuals with higher TyG had an increased prevalence of CRN. However, these associations were less pronounced in males.

### The development of the diagnostic models and the nomogram

**Figure 3:**
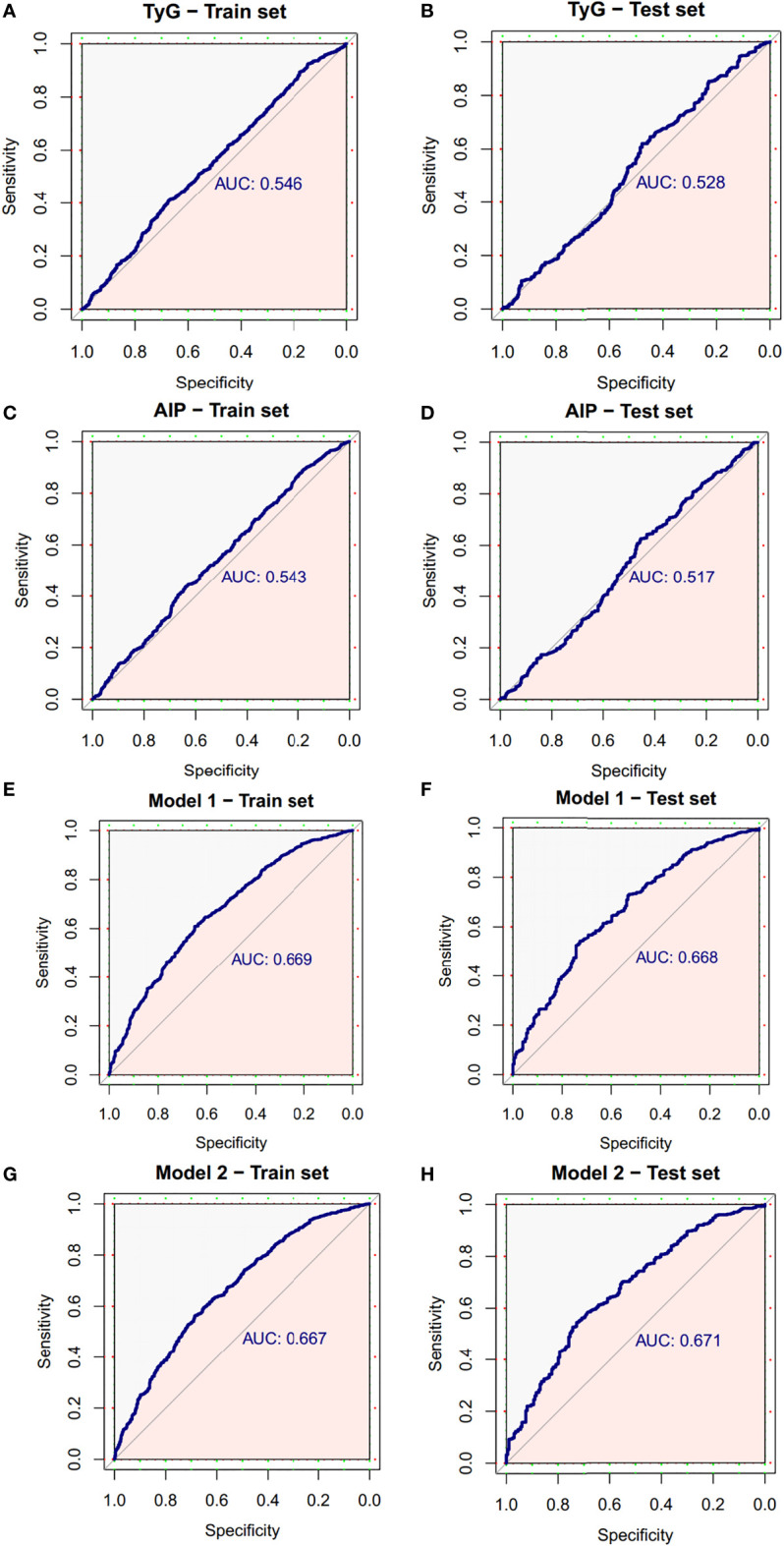
ROC curve of the diagnostic models for neoplasms. **(A)** ROC curve of the TyG index for diagnosis of neoplasms in train set. **(B)** ROC curve of the TyG index for diagnosis of neoplasms in test set. **(C)** ROC curve of AIP for diagnosis of neoplasms in train set. **(D)** ROC curve of AIP for diagnosis of neoplasms in test set. **(E)** ROC curve of model 1 for diagnosis of neoplasms in train set. **(F)** ROC curve of model 1 for diagnosis of neoplasms in test set. **(G)** ROC curve of model 2 for diagnosis of neoplasms in train set. **(H)** ROC curve of model 2 for diagnosis of neoplasms in test set. AUC, area under the curve; CI, confidence interval; ROC, receiver operating characteristic.

**Figure 4:**
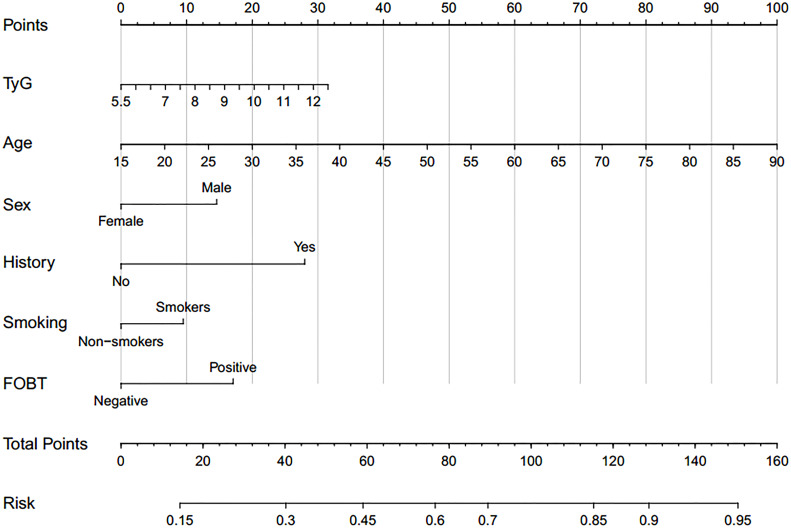
Nomogram for model 1 calculate individual neoplasm probability.

**Figure 5:**
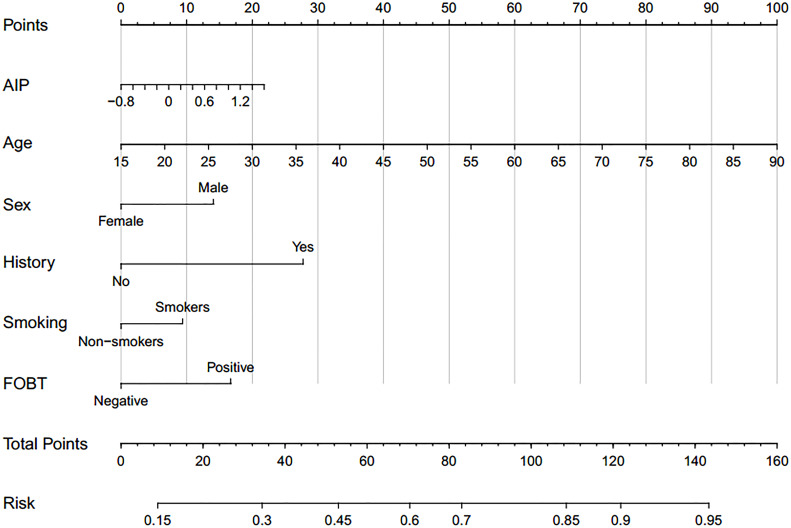
Nomogram for model 2 calculate individual neoplasm probability.

Based on TyG index and AIP respectively, and referring to the logistic regression results, the CRN prediction models were constructed based on the training set consisting of two-thirds of the total population. It was subsequently validated in the test set consisting of the remaining one-third of the population. The accuracies of the TyG-based model and the AIP-based model in diagnosing CRN are shown in **Table 3**. Model 1 (TyG, age, sex, smoking status, family history, FOBT) had an overall 61.0% sensitivity and 65.1% specificity with an AUC of 0.669 (95%CI, 0.639 - 0.698). Model 2 (AIP, age, sex, smoking status, family history, FOBT) had an overall 56.3% sensitivity and 68.7% specificity with an AUC of 0.667 (95%CI, 0.638 - 0.697). The predictive efficacies for the diagnosis of CRN were evaluated using ROC curves (**Figure 3**). All significant factors of CRN occurrence were integrated into the nomograms of model 1 (**Figure 4**) and model 2 (**Figure 5**), respectively. This was done in order to calculate the probability of an individual without cardiovascular disease having CRN.

**Table 3:** Diagnostic accuracy of TyG and AIP Index.

Model	Sensitivity	Specificity	Cut-off	AUC	95% CI
TyG					
Train Set	0.411	0.671	8.790	0.546	(0.518 – 0.574)
Test Set	0.620	0.478	8.507	0.528	(0.484 – 0.571)
AIP					
Train Set	0.438	0.637	0.161	0.543	(0.515 - 0.572)
Test Set	0.626	0.455	0.027	0.517	(0.473 - 0.560)
Model 1					
Train Set	0.610	0.651	0.653	0.669	(0.639 - 0.698)
Test Set	0.543	0.729	0.694	0.668	(0.623 – 0.714)
Model 2					
Train Set	0.563	0.687	0.669	0.667	(0.638 – 0.697)
Test Set	0.545	0.729	0.606	0.671	(0.625 – 0.716)

Model 1, TyG + age + sex + smoking status + family history + FOBT;

Model 2, AIP + age + sex + smoking status + family history + FOBT;

AUC, area under the receiver operating characteristic curves; CI, confidence interval; P < 0.05 is considered statistically significant.

## Discussion

In this retrospective case-control study, we identified both the TyG index and the AIP can be used to predict the presence of CRN in patients without CVD. In these individuals, the higher the value of TyG index, the higher the incidence of CRN (which consist of CRC and CRA). Similarly, a high AIP index was associated with a high prevalence of CRN. Furthermore, our results shown that there were differences between genders in the prediction effects of TyG index and AIP on CRN. The correlation between each of these two indicators and CRN is more pronounced in women than in men.

The medical and academic interest in the effects of the TyG index and the AIP have been increasing. The TyG index is considered a simple and useful surrogate for IR in clinical practice (21). Studies have shown that its predictive value for IR is even better than the homeostasis model assessment of insulin resistance (HOMA-IR) (22), which is the gold standard method for measuring IR. Most of the current research on the TyG index is concentrated in the field of CVD. As an independent risk factor for CVD (23), the TyG index can predict CVD risk in the general population (24). Several studies have also reported that the TyG index may predict acute coronary syndromes, symptomatic CVD and ischaemic stroke (9, 25–28). An elevated TyG index is significantly associated with a higher risk of arterial stiffness (29, 30), it is also used as a valuable biomarker for the development of type 2 diabetes mellitus (22, 31, 32) and metabolic syndrome (33). Moreover, Huihui Ren et al. identified that the TyG index was closely associated with the severity and morbidity in COVID-19 patients (34). As for AIP, it is a substitute for small, dense low-density lipoprotein-cholesterol (sdLDL-C) particles and is inversely related to the LDL-C particle diameter (35). As a marker of plasma atherogenicity, AIP has proven to be a good predictive and prognostic biomarker for cardiovascular events as well as the best predictor of hypertension and diabetes (36–38). It has also recently been proved to be a novel factor more closely related to non-alcoholic fatty liver disease than other lipid parameters in adults (39).

Although the exact mechanism causal to the correlation between the two indicators of the TyG index and the AIP and CRN has not yet been elucidated, it may be related to IR. Former studies have shown that IR is an important risk factor for the development of early colorectal neoplasia (39). It is also associated with a poorer response to anti-CRC therapy (40). IR and secondary hyperinsulinemia with an increase in insulin growth factor (IGF) levels promoted intestinal tumor proliferation through mechanisms that overlap with CRN progression (41). These include the PI3K/Akt/mTOR/S6K signaling pathway (42), the RAS-Raf-MAPK signaling pathway, and the c-JunN-terminal kinase (JNK) signaling pathway (43). As the key features in the development of IR (44), increased visceral and ectopic fat deposition can induce immune cell infiltration and macrophage activation in adipose tissue (45). This can trigger disturbances in the balance of inflammatory-regulatory cytokines, resulting in systemic persistent low-degree inflammation (46), which might promote tumorigenesis. Visceral fat can also secrete a substantial amount of heterogeneous adipocytokines (47), which further influences neoplasm development through its role in the physiological and pathological processes such as angiogenesis, cell proliferation and apoptosis (48, 49). In addition, IR contributes to subcellular component abnormalities, including lipotoxicity, oxidative stress, mitochondrial dysfunction, endoplasmic reticulum (ER) stress, and impaired calcium signaling (45, 50–52). These abnormalities might promote the development of neoplasms.

It is recognized that abnormalities of lipid metabolism are closely related to tumor cell growth, proliferation and differentiation. Previous studies have shown that both TG and LDL-C were significantly associated with an increasing prevalence of colorectal neoplasia (53), and there was an inverse correlation between HDL-C levels and colon cancer risk (54). However, compared with quantitative changes of a routine lipid profile, TyG index and AIP can better reflect the level of blood lipid metabolism in the body. Okamura et al. demonstrated that TyG index could predict the incidence of CRC, with the cut-off value of 8.272 (55). Liu T et al. found that elevated TyG index and TG/HDL-C ratio were associated with a higher risk of developing CRC (56). Stevanovic et al. demonstrated that the determination of the LDL-C particle diameter or the ratio of sdLDL-C was more meaningful in assessing the risk of CRC development than the measurement of total LDL-C concentration (57). The diameter of the sdLDL-C particle is smaller, so it is more easily oxidized to produce oxidized LDL-C (oxLDL-C), which significantly contributes to the malignant transformation of colorectal polyps (58). This suggests that the AIP can also be a useful risk marker of CRC. However, previous studies focused only on CRC, ignoring its precancerous lesions, CRA. CRA often has the tendency to undergo malignant transformation, but can be completely removed by endoscopy. Undoubtedly, far more people currently suffer from CRN than from CRC. Compared to previous studies, we enrolled a large number of patients with CRA rather than only CRC patients, and demonstrated for the first time that both the TyG index and the AIP are reliable blood-based biomarkers for CRN.

We also used a restricted cube analysis to present the effect of small changes in the TyG index and AIP on the OR value of the dependent variable as a continuous curve. Within a certain range, the OR value of CRN in females amplified with increasing AIP or increasing TyG index. Notably, no similar apparent linear or nonlinear relationship existed in the males. To the best of our knowledge, this is the first study to demonstrate such a correlation. Many researchers have demonstrated that men have higher rates of colorectal polyps and carcinoma than women (59–61), and men have more risk factors associated with metabolic disease than women. For example, men are more likely to be smokers and drinkers and have higher serum uric acid (62). Therefore, the associations of TyG index and AIP with CRN in men may be influenced by more confounding relationships, and the prediction effects of these two indicators on CRN are limited. Further prospective large-scale studies are required to confirm our findings.

In recent years, an increasing number of prognostic nomogram models for CRC have been established. However, to our knowledge, this is the first study to build predictive models for CRN based on TyG index and AIP. The models we developed had good sensitivity and accuracy, and the nomograms were constructed based on the models. Anyone without CVD can use our nomograms to conveniently assess their risk of CRN by referring to their own usual known easy parameters. Our research results can guide the targeted screening of colonoscopies in this specific population, which can increase the detection rate of CRA and prevent the occurrence of CRC. This could ameliorate a long-term prognosis and benefit more potential CRA patients, especially those who do not have medical conditions or are unwilling to undergo routine colonoscopy. On the other hand, more effective prioritization testing using colonoscopies for those with high TyG index or high AIP among non-CVD patients, could also reduce the economic and clinical burdens of current screening methods.

Nevertheless, there are several limitations need to be considered when interpreting our findings. Firstly, due to the cross-sectional study design, causality cannot be established based on the results of this single study. Secondly, although we avoided the possible effect of CVD on outcomes by selecting study subjects, we still cannot exclude the possibility of residual or unmeasured variables given the observational study design of the present analysis. Thirdly, although the number of patients in the case group is sufficient, the participants were recruited in one hospital, which may limit the generalizability of the results to other populations. More research across ethnicities and demographics is needed, and relevant prospective studies are required to confirm our findings. These additional studies will provide more information regarding the potential of TyG index and AIP in predicting neoplasms.

## Conclusion

In conclusion, the data presented in this study demonstrates that non-CVD patients with higher TyG index or AIP are more likely to have CRN. Based on the results of this innovative research, we posit that both TyG index and AIP are reliable indicators for predicting CRN in people without CVD.

## Data availability statement

The raw data supporting the conclusions of this article will be made available by the authors, without undue reservation.

## Ethics statement

The studies involving human participants were reviewed and approved by the ethics committees of Beijing Friendship Hospital, Capital Medical University. Written informed consent for participation was not required for this study in accordance with the national legislation and the institutional requirements.

## Author contributions

Conceived and designed the research: MH, HS and PL. Performed the research: MH and SY. Analyzed the data: HW and GZ. Manuscript preparation: MH. Manuscript revisions: SZ, HS and PL. All authors contributed to the article and approved the submitted version.

## Funding

This work was funded by the National Natural Science Foundation of China (82070575), Beijing Municipal Administration of Hospitals’ Youth Programme (QML20190104), Beijing Nova Program (Z201100006820147), Beijing Municipal Science & Technology Commission (Z181100001718221).

## Conflict of interest

The authors declare that the research was conducted in the absence of any commercial or financial relationships that could be construed as a potential conflict of interest.

## Publisher’s note

All claims expressed in this article are solely those of the authors and do not necessarily represent those of their affiliated organizations, or those of the publisher, the editors and the reviewers. Any product that may be evaluated in this article, or claim that may be made by its manufacturer, is not guaranteed or endorsed by the publisher.
